# Marginalised Stacked Denoising Autoencoders for Robust Representation of Real-Time Multi-View Action Recognition

**DOI:** 10.3390/s150717209

**Published:** 2015-07-16

**Authors:** Feng Gu, Francisco Flórez-Revuelta, Dorothy Monekosso, Paolo Remagnino

**Affiliations:** 1 School of Computing and Information Systems, Kingston University, Penrhyn Road, Kingston upon Thames KT1 2EE, UK; E-Mails: D.Monekosso@kingston.ac.uk (D.M.); P.Remagnino@kingston.ac.uk (P.R.); 2 Faculty of Science, Engineering and Computing, Kingston University, Penrhyn Road, Kingston upon Thames KT1 2EE, UK; E-Mail: F.Florez@kingston.ac.uk

**Keywords:** deep learning, marginalised stacked denoising autoencoders, bag of words, multiple kernel learning, multi-view action recognition

## Abstract

Multi-view action recognition has gained a great interest in video surveillance, human computer interaction, and multimedia retrieval, where multiple cameras of different types are deployed to provide a complementary field of views. Fusion of multiple camera views evidently leads to more robust decisions on both tracking multiple targets and analysing complex human activities, especially where there are occlusions. In this paper, we incorporate the marginalised stacked denoising autoencoders (mSDA) algorithm to further improve the bag of words (BoWs) representation in terms of robustness and usefulness for multi-view action recognition. The resulting representations are fed into three simple fusion strategies as well as a multiple kernel learning algorithm at the classification stage. Based on the internal evaluation, the codebook size of BoWs and the number of layers of mSDA may not significantly affect recognition performance. According to results on three multi-view benchmark datasets, the proposed framework improves recognition performance across all three datasets and outputs record recognition performance, beating the state-of-art algorithms in the literature. It is also capable of performing real-time action recognition at a frame rate ranging from 33 to 45, which could be further improved by using more powerful machines in future applications.

## 1. Introduction

Multi-view action recognition has gained a great interest in video surveillance, human computer interaction, and multimedia retrieval, where multiple cameras of different types are deployed to provide a complementary field of views. Fusion of multiple camera views evidently leads to more robust decisions on both tracking multiple targets and analysing complex human activities, especially where there are occlusions. In real world application domains, e.g., ambient assisted living, multiple cameras are often deployed to cover an extensive area. This is the case for larger indoor environments that cannot be monitored with a single sensor. The use of a multi-camera sensor is also required in applications where human behaviour is to be captured and analysed. A single camera, even when wide area coverage is adequate, is insufficient when monitoring complex human behaviour particularly in situations where in addition to position information, more detailed information about the posture, gestures and manipulation of objects is sought. It is therefore not a surprise that multiple view recognition of behaviour and action has recently attracted the attention of computer vision researchers and practitioners in the field of intelligent monitoring and smart environments.

In recent years bag of words (BoWs) [1] has become a recognised method to model and classify video data. It was borrowed from the information retrieval community [2], and is of great interest because it is unsupervised and easy to implement with problems where multiplicity of patterns is the discriminative feature, used to categorise large datasets. The method however has its disadvantages, in particular its weakness in dealing with large number of classes, the sparseness of the underlying representation and the difficulty in handling synonyms (different patterns carrying similar semantic information) and polysemies (similar patterns carrying different semantic information) [3]. Because of the above-mentioned weaknesses, stacked denoised autoencoders (SDA) have been introduced, which are a family of methods developed in the deep learning research field. SDA algorithms make use of different kinds of encoders to transform the input data, preserving a maximisation of the mutual information between the raw and the encoded information, typically using the infomax principle [4], while utilising a noise criterion for minimising the transformation error.

The paper applies the marginalised version of SDA algorithms that is computationally much less expensive, to data captured by multiple cameras (an idea easily extensible to multiple sensors), with partial overlapping fields of view. Accrued data is combined from the multiple streams with simple methods, such as concatenating the extracted information, combining the information in a sum or product. The combination techniques of information are then further improved and better formalised, using multiple kernels, as described in [5]. The proposed framework works in real-time for multi-view action recognition. The framework and variations thereof were thoroughly evaluated on a number of multi-view action datasets. The proposed framework is compared with existing and successful state-of-the-art algorithms recently published in the literature. The rest of the paper is organised as follows: Section 2 provides an overview of the related work of multi-view action recognition; Section 3 details the theory of using marginalised denoising autoencoders for robust and useful feature representation; Section 4 describes the fusion techniques used for combining multiple camera views, in particular, multiple kernel learning; Section 5 lists the experimental conditions, such as benchmark datasets used for evaluation, implementation details, and a discussion of the real-time capacity of the proposed framework; Section 6 demonstrates the results obtained from performed experiments as well as their implications; finally Section 7 concludes this work and points out some possible future directions.

## 2. Related Work

Fusion of multiple camera views often leads to more robust decisions on both tracking multiple targets and analysing complex human activities, especially where there are occlusions. Weinland *et al.* [6] introduced motion history volumes (MHV) as a free-viewpoint representation for human actions in the case of multiple calibrated, and background subtracted, video cameras. These MHV are a 3D generalisation of motion history images [7] in order to remove the viewpoint dependence. Alignment and comparison of different actions are performed using Fourier transforms in cylindrical coordinates around the vertical axis. Cilla *et al.* [8] proposed a probabilistic distributed system that fuses the posterior distribution corresponding to each camera view into a single distribution for the final decision making. Later on they applied a feature fusion approach to efficiently combine 2D observations extracted from different camera viewpoints in [9]. Mosabbeb *et al.* [10] proposed a distributed system where each camera processes its own observations, and while communicating with other cameras, they come to an agreement about the activity class. This method, which is capable of removing noise and outliers, is based on recovering a low-rank matrix over consensus to perform a distributed matrix completion via convex optimization. Burghouts *et al.* [11] used BoWs consisting of STIP features to capture motion, random forest to quantise the features into histograms, and a support vector machines (SVM) classifier as action detector. Data fusion at different stages are tested and compared. Holte *et al.* [12] demonstrated an approach that detects 4D spatio-temporal interest points and local description of 3D motion features in multi-view images, using 3D reconstructions of the actors and pixel-to-vertex correspondences of the multi-camera setup. A similar approach was presented by Yan *et al.* [13] by analysing the differential geometric properties of spatio-temporal volumes XYT for each view generated by concatenating the actor's silhouette over the course of an action. These features obtained from multiple views are then mapped to the 4D action shape.

Some approaches assume that the actor is performing the action in a pre-specified angle from the camera (view dependency). This is not true in real-world applications where the actor may not necessarily have constraints in location and orientation. Therefore, view-independent systems, which remove view dependent features, lose discriminative information for action classification. In order to resolve such an issue, Iosifidis *et al.* [14] introduced a method using a novel representation of multi-view human movement videos, which is based on learning basic multi-view human movement primitives, called “multi-view dynemes”. Fuzzy distances from the multi-view dynemes are used to represent the human body postures in a new feature space, called “dyneme space”. For solving the view identification problem, the method either uses a circular block shift procedure followed by the evaluation of the minimum Euclidean distance from any dyneme, or exploits the circular shift invariance property of the discrete Fourier transform. The resulting system produced a high human movement classification accuracy. Hashemi and Rahmati [15] proposed a hybrid method consisting of a view-dependent representation and a view-independent representation. The view-dependent representation is used to reduce the number of possible categories for each video sequence. The view-dependent representation places similar activities in the same clusters, then reduces the number of possible action's labels prior to the view-independent representation. This reduction in the number of possible actions is then fed together with the view-independent representation. It shows a significantly increased accuracy compared to other view-independent action recognition methods.

Most of the previous methods fuse pose information either before action description or post classification. Zhu *et al.* [16] employed local segments of binary silhouettes on a random forests classifier, and then applied a voting strategy of the classification results of all the decision trees to obtain the action label. Iosifidis *et al.* [17] performed single-view action representation and classification. Classifications from all the views are then combined using Bayesian learning instead of a voting system.

The above methods are computationally expensive and thus the recognition can only be performed in an off-line manner. Researchers have therefore shifted their focus onto real-time multi-view action recognition. Weinland *et al.* [18] proposed an approach to provide robustness to both occlusions and viewpoint changes, which is capable of processing over 500 frames per second while maintaining a reasonable recognition performance. Cherla *et al.* [19] used a low-dimensional feature vector obtained from the silhouette's bounding box and some spatio-temporal features. Later, dynamic time warping is used to perform recognition. This method runs at 20 frames per second. Chaaraoui *et al.* [20] presented a method, where pose representation is based on the contour points of the human silhouette and actions are learned by making use of sequences of multi-view key poses. Their method is able to process 26 frames per second, and the recognition accuracy and speed are then further improved in [21].

## 3. Feature Representation for Action Recognition

A common practice in action recognition for generating discriminative feature representation is to apply BoWs to local descriptors of motion and appearance. Local descriptors, e.g., spatio-temporal interest points (STIP) [1] and improved dense trajectory descriptor (IDT) [22], first identify some regions of interest (ROI) in the spatio-temporal space of a video, *i.e.*, interest points and trajectories. They then extract visual features, e.g., histograms of gradient, histograms of optical flows and motion boundary histograms, from each ROI to represent local appearances and motions. In order to transform such local descriptors into discriminative feature vectors that can be used by a classifier for action recognition, a codebook or dictionary must be generated. A subset of descriptors are randomly selected from the training data, and it is then fed into a unsupervised clustering method, e.g., K-Means [23], to generate a user defined number of clusters. The generated clusters are called “visual words”, and together they form the codebook. All descriptors of a video or a spatio-temporal cuboid in a video that is potentially associated with the actions of interest, are then compared to every word in codebook to compute a high dimensional histogram feature vector, by counting the numbers of closest clusters based on some metric, e.g., the Euclidean distance. As a result, instances of the actions of interest are transformed into sparse feature vectors, which can be trained with a discriminative classifier, e.g., SVM [23], for learning action models. Although BoWs originated from the area of natural language processing (NLP), it has gained great popularity in computer vision, especially for the recognition of simple and recurring actions.

### 3.1. Stacked Denoising Autoencoders

Despite the popularity, BoWs style representations suffer from their inherent over-sparsity and fail to capture world-level synonymy and polysemy [3]. The nature of codebook generation results in the majority of the dimensions of a computed histogram feature vector being zero. Such a transformation process may lead to significant information loss, so that examples of different classes become more uniform and thus insufficiently discriminative to train a classifier to distinguish between classes. In addition, BoWs representations cannot capture synonymy, that is, the virtual words which have the same connotations, implications, or reference, are considered dissimilar to each other. The BoWs representations also cannot grasp polysemy, and thus they discard the possibility of a virtual word having many different meanings. There drawbacks lead to the requirement of a trained classifier being exposed to a very large set of labelled examples, in order to gain the sufficient predictive power for new examples. It becomes more problematic when the amount of labelled data is limited and the number of classes is large, which is the trend of a lot of newly collected datasets in action recognition.

To overcome the limitation of BoWs representations mentioned above, deep learning based stacked denoising autoencoders (SDA) [24] was introduced. A traditional linear autoencoder [25] first takes an input vector **x** ∈ [0, 1]*^d^* and transforms it into a hidden representation **y** ∈ [0, 1]*^d^*^′^ using an **encoder** as,

(1) $$\text{y}=f_{\theta }\left(\text{x}\right)=\text{Wx}+\text{b}$$

and it is parametrised by *θ* = {**W**, **b**}, where **W** is a *d* × *d′* weight matrix and b is an offset vector with a dimensionality of *d′*. The mapping in Equation (1) may be replaced by a nonlinear mapping in some of the SDA algorithms. The resulting hidden representation **y** is then mapped back to a reconstructed vector **z** ∈ [0, 1]*^d^* via a **decoder** as,

(2) $$\text{z}={\text{g}}_{\theta^{\prime }}(\text{y})={\text{W}}^{\prime }\text{y}+{\text{b}}^{\prime }$$

and it is parametrised by *θ*′ ={**W′**, **b′**}. The vector is usually reconstructed following a probabilistic distribution *p*(**X**|**Z** = **z**) that may generate **x** with a high probability. In order to minimise the reconstruction error, we have to optimise the model as,

(3) $$\theta^{*},{\theta^{\prime }}^{*}=\arg\underset{\theta ,\theta^{\prime }}{\min}\frac{1}{n}\sum_{i=1}^{n}ℒ({\text{x}}_{i,}{\text{z}}_{i})=\arg\underset{\theta ,\theta^{\prime }}{\min}\frac{1}{n}\sum_{i=1}^{n}ℒ({\text{x}}_{i,}{\text{g}}_{\theta^{\prime }}\text{(}f_{\theta }\text{(}{\text{x}}_{i})))$$

where 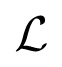 is a loss function, e.g., squared error or cross entropy if x is a binary vector. Let *q*^0^(**X**) be the empirical distribution associated with the *n* training examples, we can thus rewrite Equation (3) as,

(4) $$\theta^{*},\theta^{\prime }*=\arg\underset{\theta ,\theta^{\prime }}{\min}{\text{E}}_{q^{0}(\text{x})}\left[ℒ(\text{X},\text{Z}(\text{X}))\right]=\arg\underset{\theta ,\theta^{\prime }}{\max}{\text{E}}_{q^{0}(\text{x})}\left[\log p(\text{X}|\text{Y}=f_{\theta };\theta^{\prime })\right]$$

which is equivalent to the maximisation of a lower bound on the mutual information between **X** and **Y**. According to the *infomax principle* proposed by Linsker [4], the mutual information between an input random variable **X** and its higher level representation **Y** can be used as an important criterion for measuring the amount of information retained by the representation from the input. The higher the mutual information, the better representation is reconstructed. However, as pointed out by Vincent *et al.* [24], the criterion that representation **Y** should retain as much information as possible of input **X** by itself is not sufficient to generate a useful representation, as it is necessary to separate useful information to be retained from noise to be discarded during the reconstruction process. As a result, they propose that “*a good representation is one that can be obtained robustly from a corrupted input and that will be useful for recovering the corresponding clean input”* [24]. The reconstruction process of cleaning partially corrupted input to generate useful representation is called “denoising”. This is based on the intuitions that on one hand a higher level representation should be stable and robust under corruption of the input, and on the other hand the outcome of the denoising process is dependent on the extraction of rich features that capture useful structure in the input distribution.

A denoising autoencoder is trained by first corrupting the initial input x to get a partially destroyed version **x̃** via a stochastic mapping, e.g., **x̃** ∼ *q*(**x̃**|**x**). The corrupted version **x̃** is then mapped to a hidden representation **y** using an encoder as in Equation (1) from reconstructing a decoder as in Equation (2). The training can be performed by minimising the average reconstruction error over the training set as in Equation (4), so that **z** can be as close as possible to the corrupted input **x̃**. The corruption process is often achieved by randomly choosing *νd* components of the input **x** and setting them to zero while keeping the rest untouched, where *ν* is a predefined parameter of proportion. It is aimed to simulate the process of removing the components which are set to zero from the input. A deep network can be initialised by stacking denoising autoencoders in the same way as stacking restricted Boltzman machines [26] or basic autoencoders [27]. The training of such a deep network is usually performed in a greedy layer by layer manner, where the output of the lower layer is treated as the input the the higher layer. Once the a stack of denoising autoencoders is learnt through training, its highest layer output can be used as the input representation of a supervised learning algorithm, e.g., SVM, for further classification.

### 3.2. Marginalised SDA for Multi-View Representation via Domain Adaptation

The stacked denoising autoencoders (SDA) algorithm has been successfully applied to a wide range of problems, and yielded record performance in benchmark NLP datasets [28] even when using a simple linear SVM for the classification [29]. Although the SDA algorithm is capable of generating robust features, it has several drawbacks associated with its optimisation process. First of all, the algorithm uses stochastic gradient descent for training, making it slow and difficult to parallelise although some recent advances in a dense-matrix GPU implementation [30] and an implementation based on reconstructing sampling for sparse inputs [31] have significantly reduced the computational complexity. Secondly, it has quite a few hyper-parameters, e.g., learning rate, number of epochs, noise ratio, mini-batch size and network structure, which need to be tuned through cross validation where each run can take a considerable amount of time. Finally, the optimisation problem of the SDA algorithm is inherently non-convex, thus difficult to converge to a global optimum, and highly dependent on the initialisation for finding even a sub-optimal solution. Some of these drawbacks limit the applicability of the SDA algorithm to complex problems, e.g., the second and third drawbacks in particular for computer vision. Chen *et al.* [32] proposed the marginalised version SDA, named “mSDA”, where the noise introduced in the corruption process is marginalised. This leads to the optimal reconstruction weights being computed in a closed-form and the elimination of using back-propagation for tuning as in SDA.

Let **x**_1_,…, **x***_n_* be the inputs taken from *D* = *D_S_* ∪ *D_T_*, where *D_S_* and *D_T_* denote the source domain and target domain respectively. As in the SDA algorithm, the corruption process is performed by random feature removal, *i.e.*, setting a randomly selected subset of components to zero with a probability *p* ≥ 0. Following notations of the previous section, we denote **x***_i_* and **x̃***_i_* as the input and its corrupted version respectively. A one-layer denoising autoencoder reconstructs the corrupted inputs through a mapping **W :** ℝ*^d^* → ℝ*^d^* while minimising the following squared reconstruction loss

(5) $$\frac{1}{2n}\sum_{i=1}^{n}{\left\|{\text{x}}_{i}-{\text{W}\tilde{\text{x}}}_{i}\right\|}^{2}$$

where a constant feature that is never corrupted can be added to the input, such that **x***_i_* = [**x***_i_*; 1], in order to incorporate a bias into the mapping, *i.e.*, **W** = [**W**,**b**]. As the solution of Equation (5) depends on which components of each input are randomly corrupted, a multi-pass process over the training set is applied to reduce the variance, with a different corruption each time. Therefore, the solution of Equation (5) is equivalent to the minimisation of the overall squared loss as following,

(6) $$ℒ(\text{W})=\frac{1}{2mn}\sum_{j=1}^{m}\sum_{i=1}^{n}{\|{\text{x}}_{i}-{\text{W}\tilde{\text{x}}}_{i,j}\|}^{2}$$

where **x̃***_i,j_* is the *j^th^* corrupted version the input x*_i_* during the multi-pass process. Assuming we have a matrix **X** = [**x**_1_ , ⋯ , **x***_n_*] ∈ ℝ *^d^*^×^*^n^*, its *m* times repeated version **X̄** = [**X**,…, **X**], and **X̃** the corrupted version of **X̄**, we can rewrite Equation (5) as,

(7) $$ℒ(\text{W})=\frac{1}{2mn}\text{tr}\left[{\left(\bar{\text{X}}-\text{W}\tilde{\text{X}}\right)}^{⊤}\left(\bar{\text{X}}-\text{W}\tilde{\text{X}}\right)\right]$$

where its solution can be expressed as the closed-form solution for ordinary least squared loss [23], *i.e.*, **W** = **PQ**^−1^, where **Q** = **X̃X̃**^⊤^ and **P** = **X̄X̃**^⊤^. It can be computed as a set of linear equations without requiring any computationally expensive matrix inversions.

According to the law of large numbers, as *m* → +∞ the matrices **P** and **Q** converge to their expected values. We thus have **W** = 𝔼[**P**]𝔼[**Q**]^−1^, and 𝔼[**Q**] can be computed as,

(8) $$𝔼\left[\text{Q}\right]=\sum_{i=1}^{n}𝔼\left[{\tilde{\text{x}}}_{i}{\tilde{\text{x}}}_{i}^{⊤}\right]$$

where off-diagonal entries of the matrix ${\tilde{\text{x}}}_{i}{\tilde{\text{x}}}_{i}^{⊤}$ are uncorrupted with a probability (1 − *p*)^2^, while the probability of diagonal entries is equal to 1 − *p*. Let **q** = [1 − *p*, …,1 − *p*, 1]^⊤^ ∈ ℝ*^d^*^+1^ be a vector, where **q***_α_* is the probability of a feature *α* being uncorrupted and similar probability can be defined as **q***_β_* for a feature *β.* We also have **q***_d_*_+1_ = 1, since the constant feature should not be corrupted. If we define the scatter matrix of the original input as **S** = **XX**^⊤^, the expectation the matrix **Q** can be thus computed as,

(9) $$\text{E}{\left[\text{Q}\right]}_{\alpha ,\beta }=\{\begin{array}{ll} {\text{S}}_{\alpha ,\beta }q_{\alpha }q_{\beta } & \text{if}\;\alpha \neq \beta \\ {\text{S}}_{\alpha ,\beta }q_{\alpha } & \text{if}\;\alpha \neq \beta \end{array}$$

and the expectation of the matrix **P** can be obtained in a similar fashion as Equation (9). The reconstruction mapping **W** can thus be computed directly in a closed-form without explicitly reconstructing any single corrupted input **x̃***_i_*, and the noise is marginalised. This algorithm is known as the mSDA, which only requires to go through the data to once compute 𝔼[**Q**] and 𝔼 [**P**]. In addition, the optimisation problem is convex and thus a global optimum is guaranteed, and the optimisation process is performed in a non-iterative closed-form and therefore computationally much cheaper than the SDA algorithm. Similar to the layer-wise stacking of the standard denoising autoencoders algorithm, the marginalised denoising autoencoders algorithm can be stacked by feeding the output of the (*t* − 1)*^th^* as the input of the *t^th^* layer. Let **h***^t^* denote the output of the of the *t^th^* layer and **h**^0^ = **x** be the initial input. The training is performed in a greedy layer by layer manner, where each **W***^t^* is learned to reconstruct the previous output **h***^t^*^−1^ from all possible corruptions and the output of the *t^th^* layer becomes **h***^t^* = tanh(**W***^t^***h***^t^*^−1^) by applying a squashing function.

Chen *et al.* [33] has successfully apply the mSDA algorithm to the Amazon review benchmark dataset in NLP [28] in terms of representation learning and domain adaptation, where the mSDA algorithm outperforms all the existing state-of-the-art algorithms. It was achieved by first learning feature representation in an unsupervised manner on the union of the source and target data inputs. This is based on the observation by Glorot *et al.* [34] that sharing the unsupervised pre-training of the SDA algorithm across all the domains available is more beneficial than the pre-training performed solely on the source and target. After a mSDA is trained over all the domains, the output of all layers after squashing tanh(**W***^t^***h***^t^*^−1^) is combined with the original feature **h**^0^ to form the new representation via concatenation, where all the inputs are transformed into the new feature space. Such a transformation process involves only two meta-parameters, *i.e.*, the corruption probability *p* and the number of layers *l*, which are either pre-defined by the user or tuned through cross validation. We propose to apply a similar transformation process of the mSDA algorithm to multi-view action recognition, where video sources of the multiple camera views of the same monitored environment are temporally synchronised. The intuition of this application is that multiple views of the monitored environment can be treated as multiple domains of the same actions that are collected in different spatial perspectives. All camera views are assumed to share some structural similarities in terms of motions and appearances of the actions of interest, which are expected to be retained by the transformation process of the mSDA algorithm. As a result, we train a mSDA deep network over all the camera views and the learned deep network is then used to generate a new presentation for each camera view respectively, through the concatenation of its original input and the output of all layers after squashing as described above.

## 4. Fusion of Multiple Camera Views

Assuming the representation of each camera view is obtained through either BoWs or mSDA for the purpose of multi-view action recognition, the next step is to fuse these representations and classify an action example with respect to all the camera views into one of the action classes. Due to high dimensional nature of the input feature space, the classification stage uses discriminative models (e.g., kernel methods) to learn appearance and motion patterns of each action class, while employing various fusion techniques to combine either the visual features or the classification scores of multiple camera views for an aggregated decision making. As demonstrated in [5], various fusion techniques can be applied before, during or after the classification stage. One can combine input features of all the camera views to create a single integrated representation, which can then be fed into a classifier. Alternatively, the fusion can be achieved during the classification, where the parameters of a classifier and those of a fusion techniques can be optimised simultaneously. Finally, the classification scores of all the camera views obtained by feeding the input features of each camera view into a classifier can be combined to derive a single classification score for all the camera views. In the rest of this section, we demonstrate three simple fusion strategies as well as an advanced learning technique known as multiple kernel learning (MKL), which are used to fuse all camera views and produce an aggregated decision on the class label of an action example.

### 4.1. Simple Fusion Strategies

Let ${\text{x}}_{i}^{k}\in {\mathbb{R}}^{D}$ , where *i* ∈ {1,2,…, *N*} is the index of an input feature vector and *k* ∈ {1, 2,…, *K*} is the index of a camera view. ${\text{x}}_{i}^{k}$ is derived from the feature representation process using either BoWs or mSDA, and it is potentially associated with an action of interest. It is expected input feature vectors of all the camera views with respect to an action instance to be classified into the correct action class. We here use three simple fusion techniques [35] that combine either the input histogram feature vectors at pre-classification stage or the output classification scores of SVM classifiers at the post-classification stage as follows.

#### 4.1.1. Concatenation of Features

A simple solution is to concatenate the histogram feature vectors of multiple camera views of a spatio-temporal cuboid (an action instance) that is potentially associated with an action class, into one single feature vector ${\tilde{\text{x}}}_{i}={[\text{x}}_{i}^{1},\ldots ,{\text{x}}_{i}^{K}]$ . The concatenated feature vectors are then used as the input for training a SVM model per action class as

(10) $$f(\text{x})=\sum_{i=1}^{N}\alpha_{i}y_{i}\text{k}({\text{x}}_{i},\text{x})+b$$

where *α_i_* is the dual-form weight, *y_i_* ∈ {− 1, +1} is the label with respect to the action class, **k**(·) is the non-linear kernel function, and *b* is the bias. Thus we can compute a classification score in terms of the probability of an instance **x** being positive via a sigmoid function as,

(11) $$p(y=1|\text{x})=\frac{1}{1+\exp(-f(\text{x}))}$$

which gives the probability of the instance **x** belonging to the action class the SVM model trained for. This solution essentially transforms the multi-view action recognition problem into a single-view action recognition one through concatenating the inputs of all camera views into one.

#### 4.1.2. Sum of Classification Scores

We can also train a SVM model and compute a classification score for each camera view with respect to an action class, *i.e., p*(*y* = 1|**x**) as in Equation (11). This leads to the number of trained SVM models being equal to the number camera views multiplied by the number action classes. Input features of a new action instance are then fed to the SVM models of all the camera views with respect to an action class to produce a probability of the action instance belonging to the class in question for each camera view. All the classification scores or probabilities are then averaged to give an aggregated probability as.

(12) $$\bar{p}(y=1|\text{x})=\frac{1}{K}\begin{array}{cc} \overset{K}{\underset{k=1}{\text{∑}}}p(y=1|{\text{x}}^{k}) & \forall k\in \{1,2,\ldots ,K\} \end{array}$$

Such a fusion process basically assumes each camera view has an equal contribution to the identification of an instance belonging to one of the action classes.

#### 4.1.3. Product of Classification Scores

Alternatively if we assume that a camera view is independent of another, we can apply the product rule to the classification scores or probabilities of all the camera views as.

(13) $$\hat{p}(y=1|\text{x})=\prod_{k=1}^{K}\begin{array}{cc} p(y=1|{\text{x}}^{k}) & \forall k\in \{1,2,\ldots ,K\} \end{array}$$

The strategies of Equations (12) and (13) can be applied, due to the fact the classification scores are presented in a probabilistic form thanks to the sigmoid function in Equation (11). Generally speaking, all three basic fusion strategies ignore the difference between multiple camera views, without considering the benefit of allowing the camera views to compensate or complement each other. As a result, a more advanced fusion technique is desired.

### 4.2. Multiple Kernel Learning

MKL algorithms have been shown to be flexible and effective for learning complex problems involving multiple, heterogeneous data sources [36]. They consider multiple kernels that correspond to the multiple data sources, and combine them via a linear function such as

(14) $$K\left({\text{x}}_{i},{\text{x}}_{j}\right)=\sum_{k=1}^{K}\beta_{k}{\text{k}}_{k}\left({\text{x}}_{i},{\text{x}}_{j}\right)$$

where *β_k_* ≥ 0 and $\sum_{k=1}^{K}\beta_{k}=1$ and each kernel **k***_k_* only uses a distinct set of features. We can rewrite Equation (10) with respect to a kernel **k***_k_* as $f({\text{x}}^{k})=\sum_{i=1}^{N}\alpha_{i}^{k}y_{i}{\text{k}}_{k}({\text{x}}_{i}^{k},{\text{x}}^{k})+b^{k}$ *.* As a result, in addition to learning the *α^k^* weights and *b^k^* bias of a standard SVM model, the system also needs to learn the combination parameters *β_k_* in Equation (14). Such an optimisation problem involves two set of continuous parameters, namely “kernel parameters” (*α^k^*, *b^k^*) and “combination parameters” (*β_k_*). A popular solution is to employ a nested two-step optimisation as suggested in [36], where the first step finds the optimum of the kernel parameters (*α^k^*, *b^k^*), e.g., via quadratic programming, while fixing the combination parameters (*β_k_*), and the second step searches for the optimum of the combination parameters, e.g., via line search based gradient decent, while fixing the kernel parameters. The system keeps alternating between these two steps, until it converges to an optimal solution of both sets of parameters. Such a two-step optimisation process can be expressed in the following objective function

(15) $$\{\begin{array}{ll} \min_{\text{w},b,\text{d}} & \frac{1}{2}{\left\|\text{w}\right\|}^{2}+\sum_{i}ℒ(y_{i},f(x_{i}))+R(\text{d})\\ \text{subject}\;\text{to} & d\geq 0 \end{array}$$

where in addition to the Euclidean norm of the weight vector w of a SVM model, we have a loss function 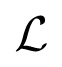 = *C* max(0,1 − *y_i_f*(**x***_i_*)) for a classification case, and a regularisation term 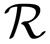 that can be any differentiable function of **d** = [*β*_1_, *β*_2_,… *,β_K_*]. Since *f*(·) is a function of **w**, the minimisation is performed over the kernel parameters of SVM models **w** and combination parameters of **d**, as demonstrated above. On one hand only mild restriction needs to be placed on the learning of **k***_K_* of the SVM model, which should be strictly positive definite for all valid **d**. This gives the flexibility of constructing different combinations of base kernels, as long as the resulting kernels are positive definite. On the other hand, the restriction of d being a non-negative orthant requires the regularisation term's derivative to exist and be continuous, *i.e.*, differentiable. Such a restriction makes it possible to apply gradient decent based optimisation techniques for the learning of combination parameters.

In [37], the authors proposed a generalised MKL algorithm [38] for object detection, where each kernel corresponds to a particular type of visual features. We adopt a similar idea, where each kernel in our framework corresponds to a particular camera view. The combination of multiple kernels is thus equivalent to the fusion of multiple camera views. The intuition behind this is that the optimal combination of parameters enables a weighted sum of all the camera views while ensuring the optimality of the kernel parameters. Such a system should be advantageous over methods merely using weighted sum of classification scores. We therefore expect that it should outperform the methods using the simple fusion techniques, due to the inherited advantage of MKL algorithms for coping with the heterogeneous nature of multiple camera views. Additionally due to the relaxed restrictions of the generalised MKL algorithm for the SVM model and regularisation term, we can implement the algorithm using existing standard libraries of SVM algorithms and gradient decent optimisation techniques.

## 5. Experimental Conditions

In order to validate our hypothesis that features representations generated by the mSDA algorithm should yield improved performance in terms of multi-view action recognition, than those derived from the BoWs approach, we conduct a series of experiments on three benchmark multi-view action datasets. Moreover, we also want to find out if the MKL (SVM-MKL) algorithm can still outperform other simple fusion techniques, e.g., concatenation (SVM-COM), sum (SVM-SUM) and product (SVM-PRD) rules, for real-time multi-view action recognition, as observed in [5]. Finally, the proposed systems in this work will be compared to the state-of-the-art algorithms tested on those benchmark datasets.

### 5.1. Benchmark Multi-View Datasets

We have chosen three well known benchmark multi-view action datasets in computer vision, namely “IXMAS dataset”, “IXMAS actions dataset” and “ACT42 dataset”. Those datasets are all focused on the recognition of a list of predefined actions of a single person in an indoor environment monitored by multiple cameras providing complementary fields of views of the scene. A multi-view action recognition system is required to take the video streams of multiple cameras and output an action class label for an instance that is defined in a particular temporal window across all the camera views.

#### 5.1.1. IXMAS Dataset

**Figure 1:**
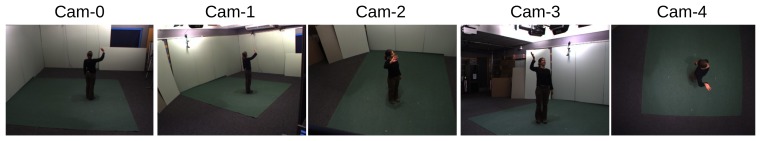
An example of all the camera views in the original IXMAS dataset.

The original IXMAS dataset was created for view-invariant human action recognition [6]. It captured 13 daily actions, each of which was performed 3 times by 12 actors. Views of 5 cameras produce video sequences at 23 frames per second and 390 × 291 resolution, as shown in Figure 1. Each actor was free to choose the location and the direction to which they face while performing the actions. As a result, the view of each camera may vary from one actor to another for different runs. We use all 12 actors and 5 cameras, and evaluate 11 actions classes as in [6].

#### 5.1.2. IXMAS Actions Dataset

**Figure 2:**
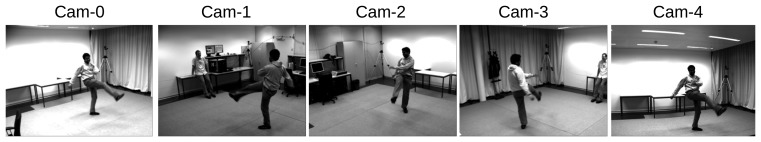
An example of all the camera views in the IXMAS actions dataset.

The IXMAS actions dataset consists of new videos of the same 11 action classes in the original IXMAS dataset [18]. The dataset was recorded with different actors, cameras, and viewpoints, so the environment settings are different from those of the original IXMAS dataset, as shown in Figure 2. There are totally 7 new actors, each of which performs 5 times per action. There overall 1148 video sequences, which are recorded in a resolution of 400 × 300 at 24 frames per second. In addition, more than 2/3 of the actions were recorded with objects in the scene partially occluding the actors in cluttered background, as main focus of the new recordings is to test a multi-view action recognition system's ability to deal with occlusions [18].

#### 5.1.3. ACT42 Dataset

**Figure 3:**
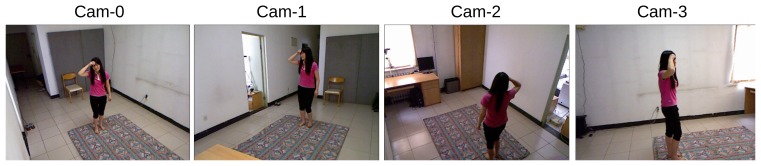
An example of all the camera views in the ACT42 dataset.

The ACT42 dataset is recorded to overcome the bottleneck of existing action recognition approaches, by providing a framework for investigating both colour (RGB) and depth information and handling action variants across multiple viewpoints [39]. All the videos are recorded in a living room environment, by 4 Microsoft Kinect cameras with a resolution of 640 × 480 at 30 frames per second, as shown in Figure 3. Please note due to the scope of this work, we only use the RGB information in our experiments, but not the depth information at all. There are 24 actors performing 14 daily actions, which results totally 6844 action instances. However only a subset of the whole dataset containing 2648 action instances is used in this work, as provided publically by the authors of [39].

All long videos that contain more than one action instance are temporally segmented into short clips, e.g., one action instance per clip, according to the ground truth. This is due to the fact, we are merely interested in the action recognition task, rather than the action detection task in which temporal localisation is required. A subject-wise leave-one-out cross validation (instances of one actor used for testing, and the remaining instances used for training) is applied to create the training set and testing set for each fold, and the reported recognition rates will be the means of all the folds.

### 5.2. Implementation Details

The improved code of [22], implemented in C++ and OpenCV, is used to generate the IDT descriptors representing motions and appearances in each video, which is downscaled to 0.5 of the original width and height (equivalent to 0.25 of the original resolution) by FFmpeg (FFmpeg by default compresses an input video when downscaling, making the video much easier and faster to compute by the IDT descriptor code.). It was clearly demonstrated in [5], the IDT descriptor yields much better recognition performance than the STIP descriptor, and the gain of computational complexity of the STIP descriptor is minor. In this paper, we therefore only use the IDT descriptor. Default parameters suggested by the original authors are used. The computation of BoWs representations uses a codebook sized {4000; 8000; 12, 000; 16, 000; 20,000}, which is quantised from 1,000,000 randomly selected descriptor features of a training set. Using different codebook sizes is to test its effect on recognition performance, since the mSDA representation often has a higher dimensionality. The codebook is then used to compute the histogram feature vectors as the inputs for the mSDA algorithm, which are transformed to a new representation. The probability of corruption *p* is chosen from {0.1; 0.2; 0.3; 0.4; 0.5} through a 5-fold cross validation in a training set, and the number of layers *l* of the deep network is set to {1;2;3;4;5}, as suggested in [32]. Choosing different numbers of layers is to test its effect on recognition performance. When the number of layers is equal to 1, the resulting network is equivalent to a multi-layer perceptron with a hidden layer. The new representation obtained by mSDA thus has a dimensionality of 4000 × 5 = 20, 000, when the input BoWs representation has a dimensionality of 4000 and the number of layers of the deep network is equal to 5. While for systems that use the BoWs representation, the computed histogram feature vectors are directly fed into a classifier. All the SVM models use ℓ_1_ normalisation and employ the *χ*^2^ kernel as

(16) $$\chi^{2}({\text{x}}_{i},{\text{x}}_{j})=2\sum_{l=1}^{D}\frac{{\text{x}}_{il}{\text{x}}_{jl}}{{\text{x}}_{il}+{\text{x}}_{jl}}$$

where *l* is the dimension index of feature vectors. All the cost parameters of SVM models are set to 100, which is found to give the overall best performance on a validation set randomly selected from the training set. The SVM code used in this work is the well known LIBSVM [40], implemented in C++. The remaining parts of the systems, e.g., the optimisation processes are implemented MATLAB Release 2014a. All the experiments are run on an Intel i7 Quad-Core CPU, 32GB RAM machine with Ubuntu 14.04 LTS operating system installed.

### 5.3. Real-Time Capacity of the Framework

Assuming the codebook of BoWs and action models are learned through training, the systems that use the BoWs representation execute the following steps at testing: *local descriptors* (1) are extracted within certain frame span; The derived local descriptors are used to compute the *histogram feature of an instance* that is potentially associated with one of the action classes (2); The histogram features are then classified by the action models to produce the *recognition results* (3). Empirically we find that the actual bottleneck of the above pipeline is step (1). The IDT descriptor code can be extracted at up to 50 frames per second, if videos are downscaled to 0.25 of the original resolution, as described in the previous section. The systems using mSDA require an additional step to generate and compute the new mSDA representations, however the optimisation of both the training and testing stages can be solved in a closed-form, *i.e.*, linear complexity. In practice, the additional step of mSDA during testing can be computed within milliseconds, due to the fact it simply computes linear functions of an input feature vector and the learned weights of mSDA from training. Therefore, it would not increase the overall runtime complexity of the corresponding systems. Since previous works in the literature have used the number of frames per second a recognition system can handle as a measure of its real-time capacity, we consider the proposed framework to meet the requirement of real-time action recognition, if the testing videos of a dataset have a frame rate less than 50.

## 6. Results and Analysis

In this section, we demonstrate the results of all the methods described in this work on the benchmark datasets listed in the previous section. The comparisons start with an internal evaluation on two important parameters, *i.e.*, the codebook size of BoWs and the number of layers of mSDA. They are followed by the comparisons between the proposed methods in this work and the state-of-the-art algorithms in the literature. The comparisons are concentrated on the average recognition rate of each method over all the action classes, as well its real-time capacity in terms of the number of frames processed per second. Various significant setting parameters or variables reported by other algorithms in the literature are also provided for the sake of comparison.

### 6.1. IXMAS Dataset

**Figure 4:**
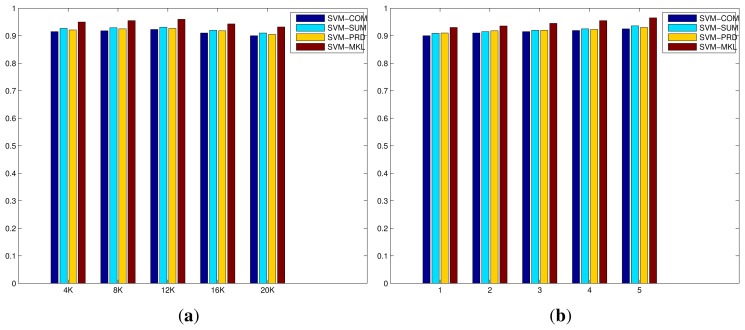
Performance comparisons on the original IXMAS dataset, of proposed methods, with respect to the size of BoWs (from 4 K to 20 K when the number of layers of mSDA is 5) and the number of layers of mSDA (from 1 to 5 when the size of BoWs is 4 K), in terms of average recognition rates. (**a**) The size of BoWs; (**b**) The number of layers of mSDA.

Figure 4 shows the evaluation of the codebook size of BoWs and the number of layers of mSDA on the original IXMAS dataset. On one hand, the increase in the codebook size results in minor improvements initially, however the recognition performances drop as the size is close to 20 K. This is due to the fact the number of selected descriptor features in the training set is limited, as the codebook size increases the number of clusters generated during the K-Means clustering increases accordingly. As a result, the average number of descriptor features per cluster has been significantly reduced, and the resulting representation become much more sparse and less discriminative. On the other hand, the increase in the number of layers of mSDA leads to slight but consistent improvements. A large number of layers results in a higher dimensional representation that is denser and more robust with respect to discrimination between different action classes. The results of all compared methods on the original IXMAS dataset are listed in Table 1, where the top half of the table consists of the offline systems while the bottom half lists the online systems with frames per second (FPS). As reported in [5], the simple fusion strategies and MKL algorithm using the IDT descriptor and the BoWs representation outperform the state-of-the-art algorithms in terms of average recognition rate. The FPS has been improved as well due to the downscaled resolution of video, which reduces the time required to compute IDT descriptors of each video. More importantly, the incorporation of mSDA further improve the overall performance of all the methods, especially the MKL algorithm has reached 0.965 at 45 FPS. This implies that the mSDA algorithm can indeed improve the basic BoWs representation by providing a more robust and useful representation, which leads to significant gains in the system's overall recognition performance.

### 6.2. IXMAS Actions Dataset

**Figure 5:**
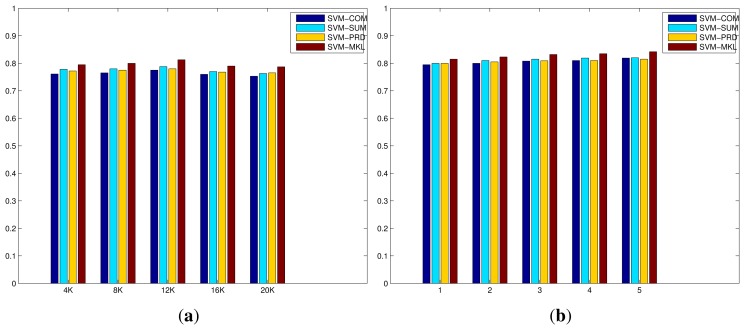
Performance comparisons on the IXMAS actions dataset, of proposed methods, with respect to the size of BoWs (from 4 K to 20 K when the number of layers of mSDA is 5) and the number of layers of mSDA (from 1 to 5 when the size of BoWs is 4 K), in terms of average recognition rates. (**a**) The size of BoWs; (**b**) The number of layers of mSDA.

Figure 5 displays the evaluation of the codebook size of BoWs and the number of layers of mSDA on the IXMAS actions dataset. Similar trends can be observed here. On one hand, the increase in the codebook size results in minor improvements initially, however the recognition performances drop as the size is close to 20 K. This is due to much more sparse and less discriminative representation when the size is larger. On the other hand, the increase in the number of layers of mSDA leads to slight but consistent improvements. This is due to the higher dimensional representation that is denser and more robust with respect to discrimination between different action classes. The results of all compared methods on the IXMAS action dataset are displayed in Table 2, the top half the table consists of the algorithms proposed in [18] using instances that are clean, *i.e.*, the occluded examples that are supposed to be more difficult to recognise are removed. While the bottom half lists all the methods applied to the entire dataset with a majority of occluded examples. All the methods described in this paper, even the basic ones using the IDT descriptor and BoWs representation outperform those in [18]. Similar trends can be observed on this dataset as well, that is, the MKL algorithm outperforms the simple fusion strategies and the new representation generated by the mSDA is more discriminative than that generated by only the BoWs, resulting significant improvements on the recognition rates. The best performance is achieved by the MKL algorithm using the representation generated by the IDT descriptor and mSDA algorithm, reaching 0.842 at 40 FPS.

### 6.3. ACT42 Dataset

**Figure 6:**
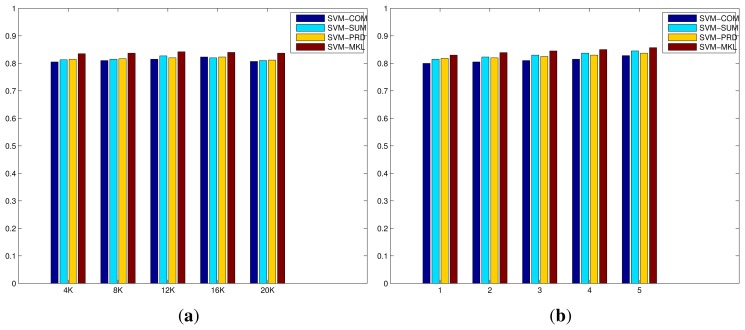
Performance comparisons on the ACT42 dataset, of proposed methods, with respect to the size of BoWs (from 4 K to 20 K when the number of layers of mSDA is 5) and the number of layers of mSDA (from 1 to 5 when the size of BoWs is 4 K), in terms of average recognition rates. (**a**) The size of BoWs; (**b**) The number of layers of mSDA.

Figure 6 demonstrates the evaluation of the codebook size of BoWs and the number of layers of mSDA on the ACT42 dataset. Similar trends can be also observed here. On one hand, the increase in the codebook size results in minor improvements initially, however the recognition performances drop as the size is close to 20 K. Again more sparse and less discriminative representation, resulted from a larger codebook size. On the other hand, the increase in the number of layers of mSDA leads to slight but consistent improvements. The higher dimensional representation is denser and more robust with respect to discrimination between different action classes. The results of all the compared methods on the ACT42 datasest are shown in Table 3, where the top half consists of the state-of-the-art algorithms in the literature, majority of which utilise both the RGB and depth data in the experiments. While the bottom half lists the methods described in this work, where only the RGB colour data is used in the experiments. Another significant difference is much increased dimensionality of the systems using the mSDA representation, due to the deep network structure and concatenated nature of the transformation process. However, since all the classifiers used are based on SVM models which are known good at handling high dimensional data, minimum impacts are caused on either the classification performance or computational complexity. In any case, although our proposed methods merely use the RGB data, thanks to richer visual features of the IDT descriptor and more robust and useful representation of mSDA, they outperform the state-of-the-art algorithms. Similarly, the recognition performance of the MKL algorithm is significantly better than the simple fusion strategies, and the systems with the mSDA representations noticeably outperform those using only the BoWs representations. The best performance is achieved by the MKL algorithm using the IDT descriptor and mSDA representation, reaching 0.857 at 33 FPS.

## 7. Conclusions and Future Work

In this paper, we extend the work of [5] by incorporating deep learning based mSDA to further improve the BoWs representation in terms of robustness and usefulness for multi-view action recognition. The resulting representations are fed into the simple fusion strategies and a MKL algorithm introduced in [5] at the classification stage. This leads to a novel framework for the application of real-time multi-view action recognition. In order to evaluate the new framework, three benchmark multi-view action datasets are used. Based on the internal evaluation, increases in the codebook size of BoWs or the number of layers of mSDA may not significantly improve recognition performance. In some cases, they even decrease the performance, because more sparse and less discriminative representations are generated. According to results, the proposed framework improves recognition performance across all three datasets, due to the incorporation of the mSDA representation, particularly the MKL algorithm using the IDT descriptor and mSDA representation outputs record recognition performance, beating the state-of-art algorithms in the literature. It is also capable of performing real-time action recognition at a frame rate ranging from 33 to 45, depending on the dataset. Since videos of the three benchmark datasets are collected at 23 FPS, 24 FPS and 30 FPS respectively, our proposed framework can still be considered real-time capable. The frame rates can be further improved by using more powerful machines in future applications. In conclusion, we have introduced a practical framework that is easy to implement and produces great performance in terms of both recognition accuracy and speed.

For future work, we want to apply the proposed framework to other vision problems with a similar setup. For instance, as depth cameras have become more and more popular and common in computer vision applications, we need to figure out a way of incorporating depth data into our existing framework. This could potentially give more flexibility to the future applications and lead to a better overall performance. In addition, it would also be beneficial to study alternative feature representation and fusion techniques, e.g., some more structure oriented fusion techniques, to further improve the recognition performance.

## Figures and Tables

**Table 1:** Comparison of all the methods in this work using IDT descriptor and BoWs or mSDA on the original IXMAS dataset, where the methods with “N/A” in the FPS column are offline systems.

**Method**	**Actions**	**Actors**	**Views**	**Reg. Rate**	**FPS**
Cilla *et al.* [8]	11	12	5	0.913	N/A
Weiland *et al.* [6]	11	10	5	0.933	N/A
Cilla *et al.* [9]	11	10	5	0.940	N/A
Holte *et al.* [12]	13	12	5	1.000	N/A
Weinland *et al.* [18]	11	10	5	0.835	500
Chaaraoui *et al.* [20]	11	12	5	0.859	26
Chaaraoui *et al.* [21]	11	12	5	0.914	207
SVM-COM (IDT + BoWs)	11	12	5	0.915	45
SVM-SUM (IDT + BoWs)	11	12	5	0.927	45
SVM-PRD (IDT + BoWs)	11	12	5	0.921	45
SVM-MKL (IDT + BoWs)	11	12	5	0.950	45
SVM-COM (IDT + mSDA)	11	12	5	0.922	45
SVM-SUM (IDT + mSDA)	11	12	5	0.936	45
SVM-PRD (IDT + mSDA)	11	12	5	0.930	45
SVM-MKL (IDT + mSDA)	11	12	5	0.965	45

**Table 2:** Comparison of all the methods in this work using IDT descriptor and BoWs or mSDA on the IXMAS actions dataset.

**Method**	**Occlusion**	**Reg. Rate**	**FPS**
Local SVM [18]	×	0.863	500
Local Weighted [18]	×	0.851	500
Local Sum [18]	×	0.825	500
Local Product [18]	×	0.815	500
Local SVM [18]	✓	0.767	500
Local Weighted [18]	✓	0.767	500
Local Sum [18]	✓	0.728	500
Local Product [18]	✓	0.689	500
SVM-COM (IDT + BoWs)	✓	0.761	40
SVM-SUM (IDT + BoWs)	✓	0.778	40
SVM-PRD (IDT + BoWs)	✓	0.772	40
SVM-MKL (IDT + BoWs)	✓	0.795	40
SVM-COM (IDT + mSDA)	✓	0.819	40
SVM-SUM (IDT + mSDA)	✓	0.820	40
SVM-PRD (IDT + mSDA)	✓	0.815	40
SVM-MKL (IDT + mSDA)	✓	**0.842**	40

**Table 3:** Comparison of all the methods in this work using IDT descriptor and BoWs or mSDA on the ACT42 dataset, where the methods with “N/A” in the FPS column are offline systems that are not reported by the original authors.

**Method**	**Feature**	**Dimensionality**	**Reg. Rate**	**FPS**
Color-HOGHOG [39]	RGB	2000	0.642	N/A
Depth-HOGHOG [39]	RGB + Depth	2000	0.745	N/A
Depth-CCD [39]	RGB + Depth	2000	0.762	N/A
DLMC-STIPs (SPM) [41]	RGB + Depth	2000	0.663	N/A
SFR [39]	RGB + Depth	2000	0.805	N/A
SVM-COM (IDT + BoWs)	RGB	4000	0.805	33
SVM-SUM (IDT + BoWs)	RGB	4000	0.813	33
SVM-PRD (IDT + BoWs)	RGB	4000	0.815	33
SVM-MKL (IDT + BoWs)	RGB	4000	0.835	33
SVM-COM (IDT + mSDA)	RGB	20,000	0.828	33
SVM-SUM (IDT + mSDA)	RGB	20,000	0.845	33
SVM-PRD (IDT + mSDA)	RGB	20,000	0.837	33
SVM-MKL (IDT + mSDA)	RGB	20,000	**0.857**	33
